# Semi-Automated Reconstruction of Neural Processes from Large Numbers of Fluorescence Images

**DOI:** 10.1371/journal.pone.0005655

**Published:** 2009-05-21

**Authors:** Ju Lu, John C. Fiala, Jeff W. Lichtman

**Affiliations:** 1 Department of Molecular and Cellular Biology, Harvard University, Cambridge, Massachusetts, United States of America; 2 Center for Brain Science, Harvard University, Cambridge, Massachusetts, United States of America; 3 Department of Health Sciences, Boston University, Boston, Massachusetts, United States of America; The University of Queensland, Australia

## Abstract

We introduce a method for large scale reconstruction of complex bundles of neural processes from fluorescent image stacks. We imaged yellow fluorescent protein labeled axons that innervated a whole muscle, as well as dendrites in cerebral cortex, in transgenic mice, at the diffraction limit with a confocal microscope. Each image stack was digitally re-sampled along an orientation such that the majority of axons appeared in cross-section. A region growing algorithm was implemented in the open-source *Reconstruct* software and applied to the semi-automatic tracing of individual axons in three dimensions. The progression of region growing is constrained by user-specified criteria based on pixel values and object sizes, and the user has full control over the segmentation process. A full montage of reconstructed axons was assembled from the ∼200 individually reconstructed stacks. Average reconstruction speed is ∼0.5 mm per hour. We found an error rate in the automatic tracing mode of ∼1 error per 250 um of axonal length. We demonstrated the capacity of the program by reconstructing the connectome of motor axons in a small mouse muscle.

## Introduction

The nervous system is comprised of a large number of neurons with extensive and specific interconnections, but the wiring diagram is largely unknown. One approach to unravel neural circuits is to reconstruct the network by imaging its cellular components. A full wiring diagram (“connectome”) would require complete reconstruction of all the connections between all cells within the network, and has only been attempted rarely, the most notable example being the nervous system of the nematode *C. elegans* done by electron microscopy [1], [2].

In recent years, with the adoption of confocal and two-photon microscopy as well as transgenic techniques to label neurons with fluorescent proteins [3], [4], it becomes possible to do connectomic studies with fluorescent microscopy. However, a main technical challenge in connectomic reconstruction is to analyze the images and delineate neural processes. A number of programs have been developed to visualize and to trace neural processes in optical image stacks, allowing the user to interactively perform or monitor the tracing. Such programs include the NeuronJ plug-in to the open-source ImageJ platform [5], as well as commercial packages such as Imaris (Bitplane AG, Zurich, Switzerland), Neurolucida (MicroBrightField, Inc., Williston, VT), Amira (Mercury Computer Systems, Inc., Chelmsford, MA), and Volocity (Improvision Inc., Lexington, MA).

These software packages do not perform satisfactorily when dealing with image stacks in which multiple neural processes branch and intertwine with each other. For instance, NeuronJ works on 2D image only, but the complexity of fasciculated nerve fibers makes it necessary to distinguish individual processes by exploring the full 3D data set. Moreover, when neural processes are closely apposed, the boundaries of such processes tend to smear into each other due to the diffraction-limited resolution of optical microscope and scattering. In this situation, the automatic or semi-automatic tracing functions provided by existing software do not guarantee correct tracing or segmentation. In addition, many of these programs do not allow segmentation tools to work on arbitrary slices. This limitation is serious because we find that reconstructing nerve fascicles is much easier from the cross-section orientation than a longitudinal one.

To facilitate the tracing of complex bundles of axons we enhanced the *Reconstruct* software [6], which was initially developed for manual segmentation of serial section electron microscopy. This platform permits the user to trace neural structures by delineating their profiles on each section of an image stack. In this way, the user can guarantee the correctness of the segmentation. The problem with this approach is that it cannot be done efficiently when large amounts of data need to be analyzed. We thus modified the software to allow faster, semi-automatic tracing of axons in image stacks. The modified program can be freely downloaded from the Yahoo group (http://tech.groups.yahoo.com/group/reconstruct_users/), which also provides a forum for user support and technical discussions. As a demonstration of the capacity of the program we reconstructed the full connectome of axons in a small mouse muscle, which required analysis of over 20,000 images.

## Results

### Image Acquisition

We imaged the axons innervating the omohyoid muscle of transgenic mice (the *thy*-1-YFP-16 line, [3]) that express cytoplasmic YFP in all motor neurons. We also imaged dendrites of cortical pyramidal neurons of transgenic mice of the *thy*-1-YFP-H line [3]. Briefly, adult mice were fixed with paraformaldehyde; the muscles were removed, post-fixed, rinsed and mounted on slides. The mouse brain was removed, post-fixed, rinsed, sliced on a vibrotome, and mounted on slides. A confocal microscope equipped with a motorized stage was used to automatically scan a montage of image stacks covering the entire area of muscle innervation. Technical details of sample preparation and image acquisition are discussed in the Methods section.

### Pre-processing of Image Stacks

Image stacks were taken on a Zeiss Pascal confocal microscope with 12-bit dynamic range to ensure sufficient signal to noise ratio when the structures to be imaged were deep or dim. The Zeiss lsm file does not have native 12-bit format, so image stacks were saved in 16-bit format, with the highest four bits being zero. Hereafter these image stacks are referred to as “XY files” or “XY stacks.”

We wrote *Matlab* scripts to preprocess these image stacks, but many of the operations are also available through other programs such as ImageJ plug-ins. As Zeiss lsm files are not among the standard file formats recognizable to the *Matlab* system, each stack (i.e., one lsm file) was converted into a series of individual 16-bit tiff files using ImageJ.

**Figure 1:**
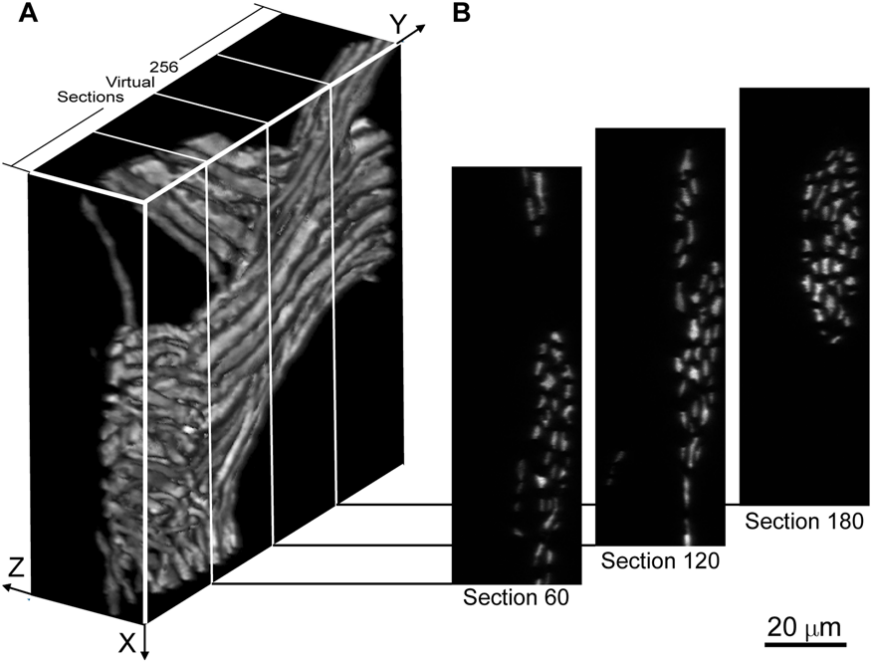
Re-sampling of a XY stack along the Y axis. A. A fluorescent image stack rendered as volume data. The raw data set contained 159 z-direction optical sections. Each section is a 1024×1024 image. X-Y pixel size: 0.10×0.10 µm; z-step size: 0.20 µm. B. En face view of three virtual sections generated by resampling the stack in A along the Y-axis at positions schematically indicated by white lines in A.

XY files were first converted to 8-bit, in which the dimmest pixel in the stack was mapped to value 0 and the brightest pixel in the stack mapped to value 255. In *Matlab* this was performed with the *imadjust* function. Each XY stack (Figure 1A) was then loaded in *Matlab* as a 3D array, and re-sampled along either the X-axis or the Y-axis with standard array manipulation functions in *Matlab*. The axis for re-sampling (the preferred axis) was chosen so that the majority of axons in the stack would appear in their cross-sections orthogonal to their long axes (Figure 1B). Although images were taken at the Nyquist limit, we found that in many stacks the structures to be traced were not very densely compacted or highly complicated, and a lower resolution sufficed for reconstruction. In these cases we used a bicubic interpolation algorithm in *Matlab* (the *imresize* function with ‘bicubic’ option) to downsize the XY stacks before re-sampling to reduce the number of sections to be analyzed without losing the ability to track individual axons. This downsizing operation has two additional advantages: it in effect applies a mean filter to the original image and thus reduces the noise, and each re-sampled image will have square pixels as required by *Reconstruct*, since the original Z step size was twice that of the X-Y pixel size.

### Semi-automatic Tracing of Axons

The original platform of the *Reconstruct* program allows a user to trace objects in serial sections by manually drawing the outline of each object on each section, which is time-consuming. We modified *Reconstruct* to enable semi-automatic tracing of axons using a region-growing algorithm called *wildfire*. The *wildfire* tool can be quickly guided by user input in an intuitive way, and generates a boundary enclosing the contiguous area of an axonal profile, while ignoring the outer halo of disjoint, scattered bright pixels common to confocal data.

The *wildfire* tool in *Reconstruct* allows the user to initiate region growing by selecting a “seed” pixel by a mouse-click. Region growing expands outward from the seed pixel to all 4-connected neighboring pixels (i.e., pixels with coordinates (*x*+1, *y*), (*x*−1, *y*), (*x*, *y*+1), and (*x*, *y*−1), given the seed pixel coordinate (*x*, *y*)) that fail to satisfy user-specified stopping criteria based on hue, saturation and/or brightness. These pixels at which growth does not stop then serve as new “seeds” for the next iteration of growth. Region growth stops when all pixels at the frontier of growth satisfy the stopping criteria and thus provide no new seed. Once the growth process stops, a labeled boundary of the region is generated by tracing clockwise around the outermost frontier of pixels. The user can block region growing by using the mouse to define temporary boundaries.

When there are many fragments of the same structure appearing on the same section (e.g., at the highly branched neuromuscular junction), it is desirable to be able to trace all these fragments on a single section with a single command rather than requiring the user to click inside every profile. We thus implemented a feature to allow the user to specify a rectangular region by dragging the mouse across the image. The *wildfire* tool then traces all noncontiguous profiles in the rectangle using the region growth algorithm and the same stopping criteria. A user-specified size threshold is used to block the generation of outlines around isolated pixels.

**Figure 2:**
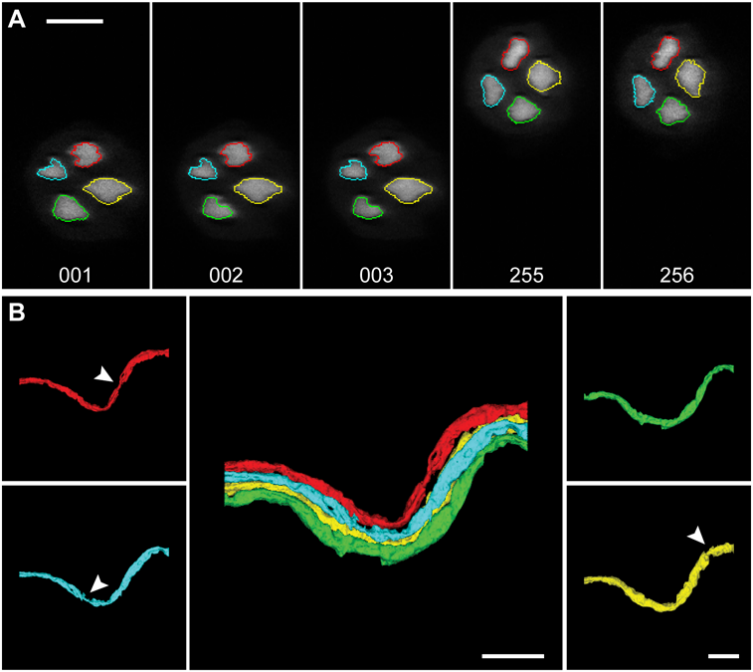
Reconstruction of an un-branching nerve fascicle. A. Axons in the nerve fascicle were traced out across multiple sections. Traces on the first 3 sections and the last 2 sections of the stack are shown. Scale bar: 10 µm. B. Traced axons were rendered in *Reconstruct*. Constrictions in the axons (arrows) represent nodes of Ranvier. Scale bars: 20 µm.

Region growing is extended to serial sections by using the centroid of each trace to locate a seed pixel for *wildfire* on the next section. Successful region growing is thus repeated on successive sections automatically (Figure 2A). To control this propagation, constraints are imposed based on the knowledge that biological structures like axons typically do not make abrupt turns, or suddenly enlarge or shrink; therefore the cross-sections of the same object on successive sections should be similar to each other in location, shape and size. The area of each new region is compared with that on the previous section; if the two areas differ by a user-specified percentage (e.g. 50%), or the area is too small (e.g. less than 10 pixels), the propagation will stop. The user can re-initiate the *wildfire* tracing with a mouse click. The stopping criteria (such as the hue, saturation or brightness thresholds) can also be modified to improve performance after a stop. Another constraint is that different axons cannot overlap with each other. The user can set a minimal distance between axons (e.g., 3–5 pixels), and the region-growing procedure will stop when it reaches such “forbidden zones” defined by the boundaries that have been already traced.

**Figure 3:**
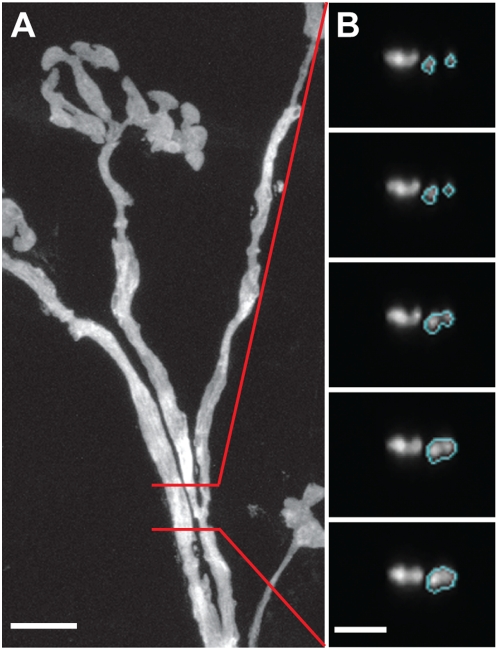
Example of a branching axon. A. Maximum intensity projection of an axon that branched between the two red lines. Scale bar: 10 µm. B. The axonal branching point shown in cross-sections. Scale bar: 5 µm.

The program also typically stops at branching points. Axons branch only at nodes of Ranvier, which show characteristically smaller diameters than the internode regions (Figure 2B and 3A) and subsequent emergence of two or more distinct profiles (Figure 3B). By recognizing this characteristic morphology, the user can easily re-initiate tracing on each of the branches with mouse clicks. Automatic tracing can continue with each of the branches, either one at a time or all together simultaneously.

**Figure 4:**
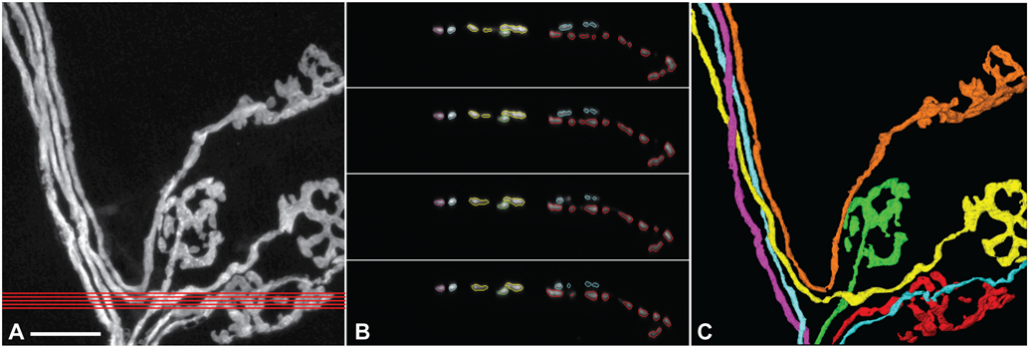
Example of axons traveling perpendicular to the preferred axis. A. Maximum intensity projection of a stack in which some axons lay parallel or oblique to the cross-sections. Red lines indicate the orientation as well as the position of virtual sections shown in B. Scale bar: 20 µm. B. Virtual sections of the region between the red lines in A. Part of the yellow axon traveled parallel to the virtual sections and appeared as multiple disconnected segments in successive sections. C. 3D rendering of all axons in this stack.

Another difficulty lies where axons do not go parallel to the preferred axis of re-sampling. Sometimes axons fan out and go in all directions, and no matter which axis is chosen for re-sampling, there are always some axons (or parts of axons) that go almost perpendicular to it (Figure 4A). An axon in this category does not appear as a single ellipsoid on each cross-section, but often as a series of fragmented, elongated pieces with variable lengths (Figure 4B). Based on contiguity of the same axon across multiple sections, the user can trace all the sectioned pieces belonging to the axon by initiating *wildfire* on each piece.

The reconstruction procedure described above generates multiple 2D contours of each axon throughout the stack. These contours can be rendered as 3D objects in different ways for visualization and subsequent merging (for details of the rendering methods provided by the program, see [6] and the manual of the *Reconstruct* program provided at the download site).

**Figure 5:**
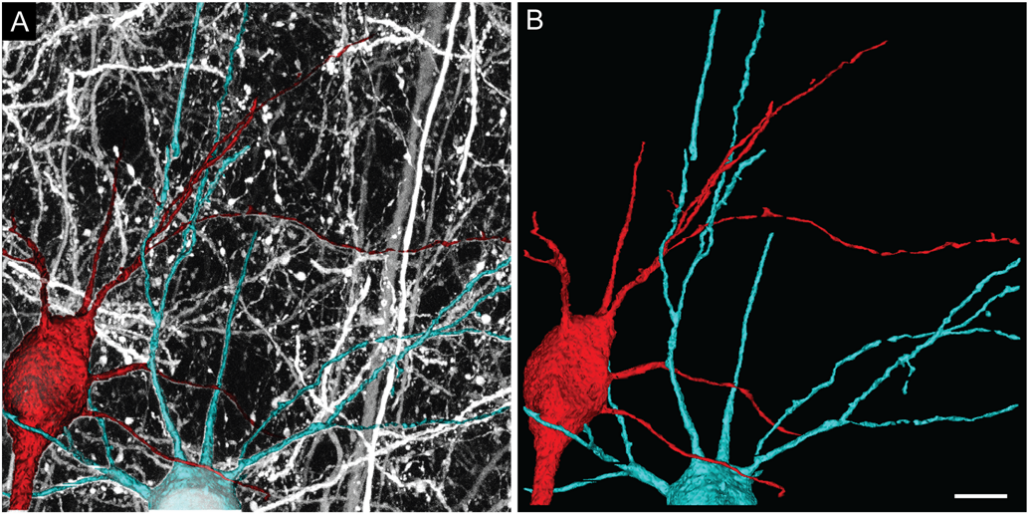
Reconstruction of dendrites of cortical pyramidal neurons. A. Dendrites of two pyramidal neurons were reconstructed together with part of the cell bodies from an image stack that contained many en passant neural processes. Reconstructed neurons were superimposed on the maximum-intensity projection of the entire image stack. B. 3D rendering of the two reconstructed neurons. Scale bar: 10 µm.

We also tested this algorithm in tracing dendrites of cortical pyramidal neurons in a YFP-H transgenic mouse [3]. We traced the dendrites of two nearby neurons from their somata within a confocal stack. As shown in Figure 5, we could clearly distinguish the processes belonging to these two neurons from *en passant* processes of other cells.

### Concatenating Adjacent Stacks

**Figure 6:**
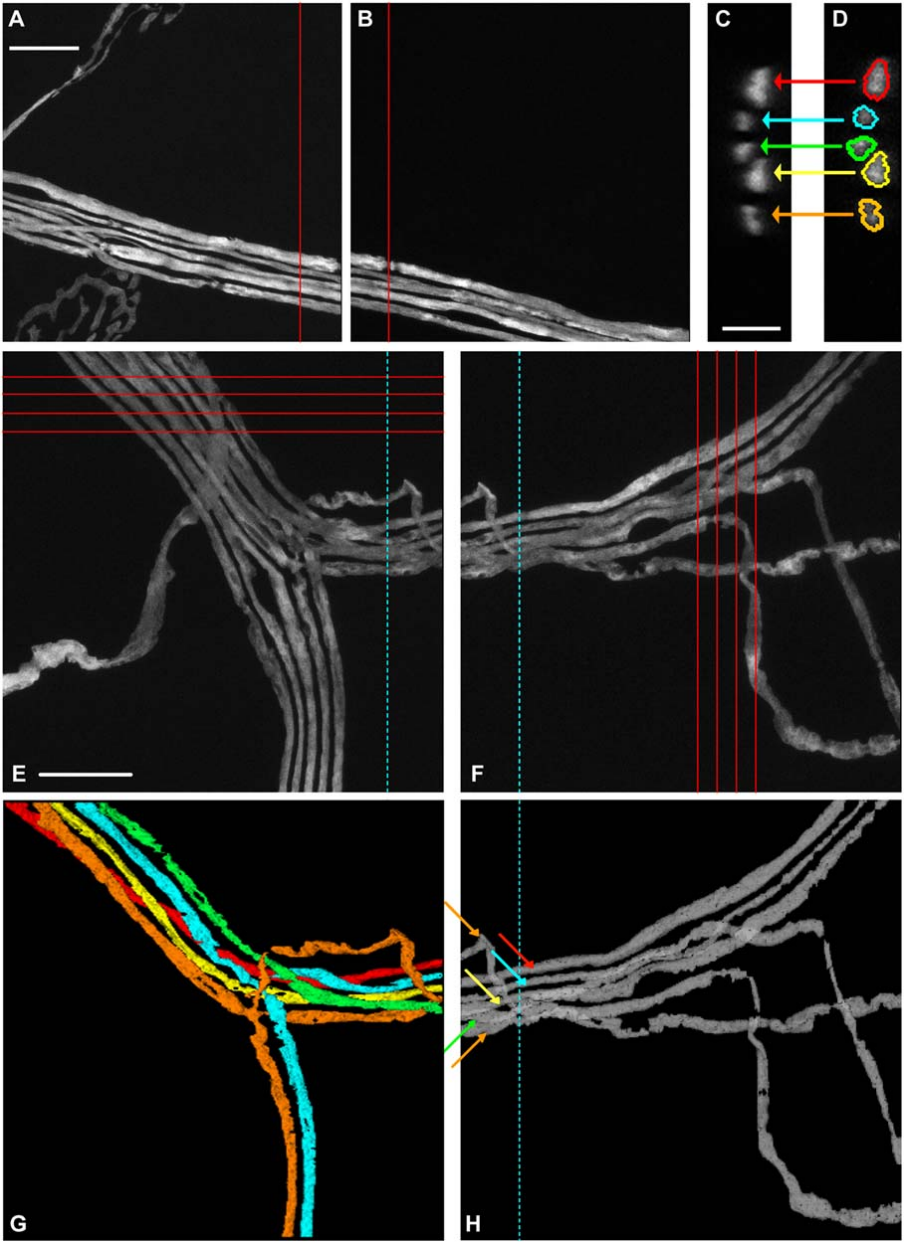
Concatenation of adjacent stacks. A–B. Two adjacent stacks with the same preferred axis. Red lines: orientation of virtual sections. Scale bar: 20 µm. C–D. Cross-sections of the same axon in the two stacks were almost identical on corresponding virtual sections. C: section 231 of stack A. D: section 001 of stack B. Scale bar: 5 µm. E–F. Two stacks that overlapped but had different preferred orientations. Red lines: orientations of virtual sections. Cyan dotted lines: boundary of the overlapping region. Scale bar: 20 µm. G–H. Corresponding axons in E and F were identified using 3D rendered images based on their morphologies and relative positions in the overlapping region. G: Reconstruction of axons in E. Axons that did not go through both stacks were omitted for clarity. Arrows in H point to axons corresponding to the reconstructed ones in G. Arrow colors are matched to axon colors in G.

Although the *Reconstruct* software can montage multiple images in each section, the fact that different stacks were re-sampled in different directions made it necessary to use *Reconstruct* to trace one stack at a time. Within each stack, each distinct axon is recognized by the unique name the user assigns to it. However, axons go across multiple image stacks and it is important to make sure that the same axon is given the same name and color in all the stacks it traverses. If two adjacent stacks have the same preferred direction (Figure 6A and B), concatenation can be easily done through inspection of a single section in the overlapping region. For example, section 231 of the left stack (Figure 6C) is almost identical to section 001 of the right stack (Figure 6D). If axons in the right stack have been traced out, direct comparison of the two sections can unambiguously determine the correspondence between each axonal profile in the left stack with its counterpart in the right stack, and tracing the left stack can proceed with known axonal identity.

When the two adjacent stacks have different preferred directions (Figure 6E and F) it is no longer feasible to directly compare the sections in the overlapping region, as none of the re-sampled sections appear identical. The solution is to first reconstruct the two stacks independently, and then match the corresponding axons in 3D rendered view, as *Reconstruct* allows arbitrary rotation of rendered objects. For instance, axons in the left stack (Figure 6G) are rendered, using a unique color for each distinct axon (axons that do not continue into the right stack are omitted from the rendering for clarity). The correspondence between identified axonal segments in the left stack and the reconstructed but unidentified segments in the right stack (Figure 6H, in gray) is easily established. Subsequently the names and colors of axonal segments in the right stack can be changed in *Reconstruct* to be consistent with that in the left stack.

### Assembly of Montage

The reconstructed individual stacks need to be assembled into a full montage covering the entire sample. We first used Photoshop to manually montage the maximum intensity projection (MIP) images of all stacks (in our case monochromatic images) to provide a reference map. The overlap between adjacent stacks enables accurate alignment of the MIP images into a complete montage. This reference map facilitates obtaining the correct magnification for reconstructions from different stacks.

For each reconstructed stack, all axons were rendered in 3D in *Reconstruct*. The 3D rendering was rotated by a suitable angle to make it *en face*, i.e., viewed in the original XY orientation, and exported as a bmp or jpeg image. The rendering of all axons in the stack collectively was aligned onto the monochromatic montage with suitable resizing. Then each axon in the stack was rendered one by one and saved separately. These individual images were superimposed onto the montage subsequently, with one Photoshop layer per image. The collective rendering now serves as the reference for the alignment of individual axons. The reason to use a separate Photoshop layer per axon is to allow the user to turn on or off any axon from the view later. This procedure was repeated for each stack until the entire montage was aligned and colored. Then all layers belonging to the same axon were collapsed in Photoshop, allowing each axon to occupy a separate layer. In order to make the appearance of individual axons more distinguishable, we used the Photoshop magic wand tool to select one axon at a time on its layer, and used the paint bucket tool to fill its interior with a distinct color.

**Figure 7:**
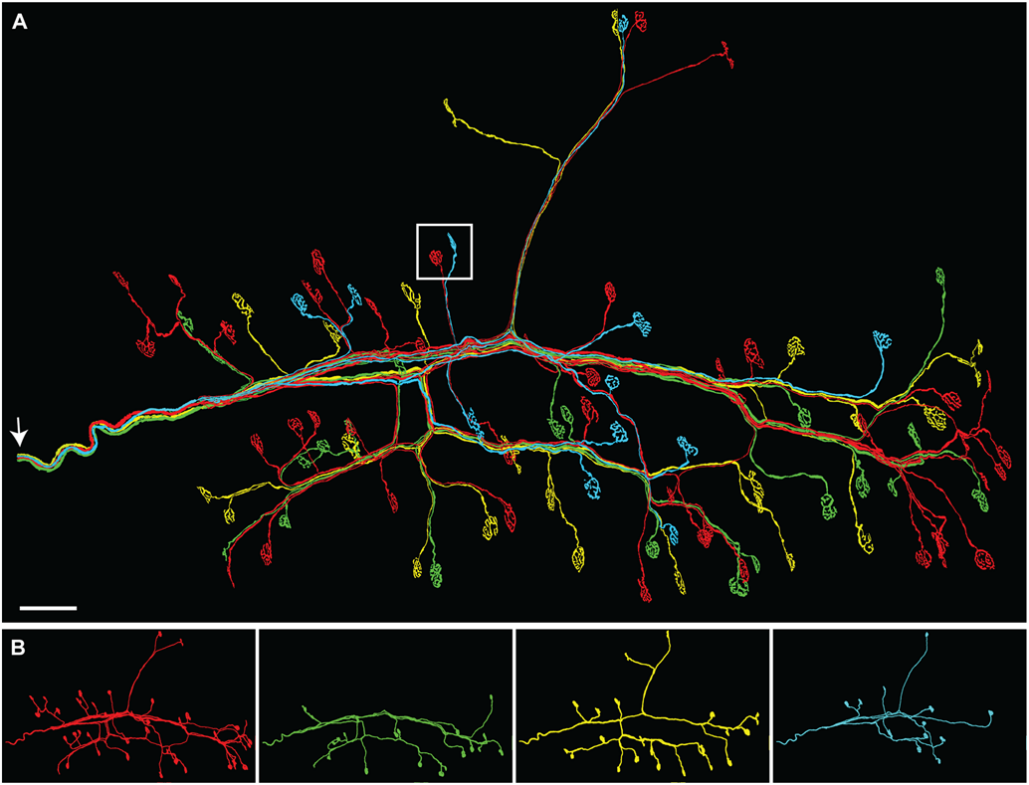
Full montage of reconstructed axons. A. The entire connectome of an omohyoid muscle with 4 axons and 96 neuromuscular junctions. The white square indicates the size of one single image stack relative to the full montage of 168 stacks. Arrow: the entry point of the nerve into the muscle. Scale bar: 100 µm. B. Each axon in the connectome shown separately. Motor unit size: red (41), green (22), yellow (21), cyan (12).

The procedure described above produces a 2D montage of the entire sample (Figure 7). However, as we already have the full 3D reconstruction of each stack, and as *Reconstruct* can export 3D rendering of objects in VRML formats, it should also be possible to do the alignment using VRML objects in a 3D modeling program.

### Evaluation of the reconstruction method

We evaluated the effectiveness of the reconstruction method presented above in terms of the reconstruction speed and the error rate. The reconstruction speed depends on the complexity and layout of the axonal bundle, as well as image quality (e.g., signal to noise level). A stack that contains axons that are homogenously labeled, well separated, and imaged with high signal to noise ratio can be reconstructed without much user intervention, and the reconstruction speed approaches ∼4 mm per hour. In this case most of the time is consumed by the delay (a fraction of a second) after generating a contour on each section, which is deliberately introduced to enable the user to see the result clearly. However, stacks that contain axons that “bleed” into each other, or are dimly labeled, or travel along non-preferred directions, take much more human intervention and manual reconstruction to complete, and the speed is consequently much slower. According to our experience, the average reconstruction speed for the whole muscle sample is ∼0.5 mm per hour [7].

The error rate of segmentation algorithms is usually determined by comparing the results of the automatic segmentation and that of manual segmentation. For our semi-automated approach, however, it seems that the usual metric of “error rate” is not appropriate, because the program does not proceed all by itself and let the user correct the answers afterwards. In fact, the design of the semi-automated feature is to allow the user to discover any error in the *wildfire* segmentation as soon as it emerges, and correct it, so that the error does not propagate. Therefore, we believe that a better metric is the rate at which the semi-automated reconstruction process requires user intervention. This rate not only gives an estimation of the reliability of the automated processing, but affects the speed of reconstruction as well.

The rate of intervention depends critically on the complexity of the data. We thus used stacks of different complexity to estimate the rate of intervention. We reconstructed 9 axons from 2 “simple” stacks (Figure 2 and Figure 6B), and 6 axons from a “complex” stack (Figure 1A). Axons in the “simple” stacks have relatively homogeneous intensity and are well separated from each other. Axons in the “complex” stack are more variable in intensity and occasionally get very close to each other. We further classified user interventions into “stops” and “errors”. “Stops” refer to the fact that the program automatically stops tracing and waits for user re-initiation. We identified 3 broad categories of events that can lead to stops: (1) the topology of the axonal profile changes (e.g., branching points), which makes the location and size of the axonal profiles on the subsequent section differ significantly from that on the previous section; (2) the intensity and/or size of the axonal profile changes sufficiently; (3) the shape of the axonal profile becomes concave (this may happen when a large mitochondrion is present, which is not labeled and thus shows up as a dark hole in the axon) and thus the “seed” pixel falls outside the contour of axonal profile and fails to initiate the new round of tracing. “Errors” refer to the case in which the program erroneously segments but does not stop by itself. A summary of rate of intervention is given in Table 1 (unit: number of occurrences per 100 µm of axons reconstructed). We excluded axons that were very dim and those that were very tightly intertwined with other axons from the analysis above. In these cases manual tracing would be preferred, given the large number of times the automated algorithm would require human intervention.

**Table 1:** Rate and Reasons of User Intervention in Reconstruction.

Stack	Topology Change	Size/Intensity Change	Initiation Failure	Errors
**Simple** (n = 9)	1.3	3.5	0.8	0
**Complex** (n = 6)	2.2	7.9	1.0	0.4

Unit: number of occurrences per 100 µm of axons reconstructed.

## Discussion

In this paper we introduced a method for large scale reconstruction of neuronal processes from fluorescent image stacks. The processes are imaged at the diffraction limit with a confocal microscope. Images are pre-processed to remove noise and re-sampled so that tracing of axons can be performed along a convenient orientation (X, Y or Z axis) which shows the cross-sections of the majority of axons. A semi-automatic program based on the infrastructure of *Reconstruct* was developed and applied to the tracing of individual image stacks. The program employs a region-growing algorithm, and uses the centroid of an existing axonal contour as the seed for region-growth on the next section in order to proceed automatically. For a non-branching, well segregated axonal process the program can automatically segment it through the entire stack (e.g., 256 sections) without interruption or human intervention in 2–3 min (∼4 mm per hour). The program stops when ambiguity arises, and the user has full control over the segmentation process. A full montage can then be assembled from the reconstructed stacks.

Several factors must be considered in the design of a program for image reconstruction from large data sets. Obviously, it is desirable to automate as many operations as possible. For connectomics, automation is especially important, as the amount of data to be processed is usually large, and manual segmentation is one of the main bottlenecks. On the other hand, the variability and complexity of the structure of the objects to be reconstructed means that some user monitoring and intervention is necessary. A user-friendly interface is thus required. If online user monitoring is required, the algorithms used in the automatic segmentation cannot be too time-consuming. This is the reason that we adopted the fast and simple region-growing algorithm based on pixel values for segmentation. If the strategy is to first go through the data automatically and then let the user validate and correct the results, the automatic processing can employ more sophisticated and computationally expensive algorithms. Many image processing algorithms, such as live wire [5], active contour or snake [8], level sets [9], Kalman filter and optical flowlevel sets [10], wavelet-based segmentation [11], and kernel-based tracking [12], [13], have been proposed for tracing 2D and 3D filamentous objects such as axons and dendrites.

The *Reconstruct* program processes images in an essentially 2D manner. Therefore one particular orientation must be selected and maintained for each stack at the re-sampling step. When objects within the stack assume very different main axes, this requirement of a single orientation leads to some inconveniences for objects along non-preferred directions. Manual segmentation is often necessary for such objects as discussed above. An alternative strategy would be to dynamically re-orient and re-sample the stack along the local preferred direction as tracing proceeds. This will ensure that at each step, the object is processed on its cross-section, which is advantageous for segmentation. This approach, however, is computationally more demanding, and remains to be fully explored.

The reconstruction method presented in this paper is applicable to the analysis of branching, tubular structures (e.g., neural processes of both peripheral and central nervous system, blood vessels, lung airways) imaged with fluorescent microscopy techniques that can obtain volumetric data (e.g., confocal and two photon microscopy). We also expect that the reconstruction method is compatible with fluorescent image stacks taken by Array Tomography [14], Selective Plane Illumination Microscopy [15], as well as ultramicroscopy [16]. Images taken with electron microscopes, however, may not be well segmented by the semi-automated algorithm presented here, because in such images neural structures are typically distinguished by their enclosing membranes, which show up as closed contours, and there is no universal intensity-based distinction between “signal” and “background.” Of course, these images may still be analyzed manually with the *Reconstruct* program as reported previously [6]. In summary, there is no intrinsic restriction on the type of tissue preparation; as long as the structures of interest can be distinguished from the background by their intensities (or hue/saturation) in the image stack, the semi-automated segmentation can be utilized.

## Materials and Methods

### Sample preparation

All animal experiments were conducted according to protocols approved by Harvard University Institutional Animal Care and Use Committee (IACUC). Transgenic mice of *thy*-1-YFP-16 line (Feng et al. 2000, now available from the Jackson Lab, Bar Harbor, ME) were used throughout these studies. Young adult mice (∼30 days old) received an intraperitoneal injection of 0.1 ml/20 g ketamine-xylazine (Ketaset, Fort Dodge Animal Health, U.S.A.), and were perfused transcardially with 4% paraformaldehyde (PFA) in 0.1 M phosphate-buffered saline (PBS; pH 7.4). For the muscle preparation: the omohyoid muscle along with a short length of the innervating nerve was removed, post-fixed in 4% PFA for 30 min, rinsed in PBS (room temperature, 30 min×2), and then mounted on slides with the Vectashield mounting medium (Vector Laboratories, Burlingame, CA). Mounted slides were slightly squeezed between a pair of small magnets over night to flatten the tissue so that the distance from tissue surface to the coverslip was roughly constant. For the brain slice preparation: the whole brain was removed from the skull, post-fixed in 4% PFA over night, rinsed in PBS (room temperature, 30 min×2), sliced at 50 or 100 µm thickness with a vibrotome (Leica VT1000S), and mounted on slides with the Vectashield mounting medium.

### Confocal Imaging

Samples were imaged using a confocal laser scanning microscope (Zeiss Pascal, Carl Zeiss, Jena, Germany) equipped with a motorized stage. We used a 63×1.4NA oil-immersion objective and optically zoomed-in by a factor of 1.5. YFP fluorescence was excited with a 488 nm Argon laser and detected through a band-pass emission filter of 530–600 nm. Images were sampled at the Nyquist frequency in the x-y direction (pixel size = 0.1 µm) and over-sampled by a factor of ∼2 in the z direction (z-step size = 0.2 µm), with 12 bit dynamic range. According to the well-known sampling theorem, a signal that contains data at maximal frequency *f*_max_ must be sampled at least at frequency 2*f*_max_ to ensure that the signal can be accurately recovered from the sampling [17]. This minimal sampling frequency is called the Nyquist frequency. In the imaging system, the maximal spatial frequency is determined by the resolution of the microscope, and for the particular imaging condition we used the resolution is ∼0.2 µm in the x-y plane, and ∼0.75 µm along the z axis [18]. Thus we used the optical zoom feature of the microscope to obtain pixel size that was at the corresponding Nyquist frequency. The motorized stage was controlled by the *MultiTimeZ* macro (developed by Carl Zeiss) to set up the coordinates and imaging conditions for each stack in the montage. Adjacent stacks had 10% overlap to guarantee the precision of later alignment and tracing.

### Image Processing

Image stacks were pre-processed with ImageJ (NIH, http://rsb.info.nih.gov/ij/) and custom-written programs in *Matlab* (The MathWorks, Inc.), and reconstructed with *Reconstruct* (http://synapses.clm.utexas.edu/tools/reconstruct/reconstruct.stm). Final assembly into a complete montage was done with Adobe Photoshop (Adobe Systems Inc.). See Results section for details.
